# Tracing Aquatic Macrophyte Development in Nansi Lake, Northern China's Largest Freshwater Lake: Plant Macrofossils From 1855 to Present

**DOI:** 10.1002/ece3.70878

**Published:** 2025-01-23

**Authors:** Qinghui Zhang, Yufei Wu, Liwei Yang, Zekun Li, Zonglei Li, Yuying Yang, Shiyue Chen, Enfeng Liu

**Affiliations:** ^1^ College of Geography and Environment Shandong Normal University Ji'nan Shandong China; ^2^ School of City and Environment Jiangsu Normal University Xuzhou Jiangsu China; ^3^ Academic Affairs Division of Xuzhou College of Industrial Technology Xuzhou Jiangsu China

**Keywords:** aquatic macrophyte, Lake Nansi, multiple factor analysis, plant macrofossil, restoration, the Yellow River (Huanghe River)

## Abstract

Examining the impacts of natural and anthropogenic influences on aquatic macrophytes in shallow lakes is crucial for their effective restoration and management. However, there is a lack of direct evidence regarding past species composition or detailed and continuous evidence of recent changes in aquatic macrophyte communities. This study utilized plant macrofossil remains deposited in the sediment, combined with macrophyte surveys from 1983 to 2010, to reconstruct the historical changes in the macrophyte community over approximately 160 years in Lake Weishan, a sub‐lake of Lake Nansi located in the lower Yellow River (Huanghe River) Basin, northern China. Approximately 54.3% of the species historically recorded at the core site were identified through macro‐remains analysis, including five previously unrecorded submerged taxa ( *Myriophyllum verticillatum* , *Ranunculus trichophyllus* , *Chara* sp., *Nitella* sp., and *Vallisneria spinulosa*) discovered during monitoring surveys. The findings revealed four major shifts in the macrophyte community: A transition from a swampy environment dominated by emergent/wetland plants (ca. 1855–1875) to an expanded water body characterized by a rapid proliferation of submerged macrophytes (ca. 1875–1910), followed by mass disappearance of macrophytes (ca. 1910–2005) and subsequent significant resurgence (after 2005). Multiple factor analysis was employed to investigate the correlation between these shifts and changes in paleolimnological indicators (invertebrates, geochemistry, and grain size), as well as documented records related to hydrology, climate changes, and human activities. The results confirmed our hypothesis that climatically and anthropogenically induced hydrological alterations were likely the primary drivers influencing macrophyte composition alteration and succession dynamics in the lake. This study highlights the potential use of plant macrofossils for reconstructing long‐term changes in macrophyte community components, abundance assessment, and ecosystem health evaluation within the lower Yellow River region. To effectively address persistent challenges such as water diversion and climate change, we propose integrating paleoecological methods into standard ecological monitoring protocols employed for water ecological quality assessment.

## Graphical abstract

This study presents a detailed plant macrofossil record of a floodplain lake in the lower Yellow River (Huanghe River) Basin, which has undergone significant channel modifications and catastrophic floods. The lake also serves as an impoundment along China's Eastern Route of the South‐to‐North Water Diversion Project. The results cover time periods from before the formation of unified Lake Nansi to present times, including the period following the implementation of SNWDP‐ER. These records offer a comprehensive historical account of how aquatic macrophytes have responded to natural changes and human activities, encompassing climate variations and anthropogenic influences.

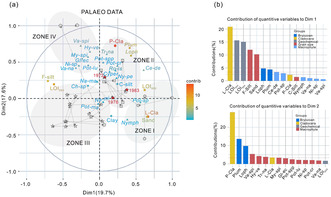

## 1 Introduction

Shallow lakes, serving as pivotal nodes in the intricate interplay of diverse environmental elements on terrestrial surfaces, not only constitute crucial water resources for human survival but also play a vital role in maintaining regional ecological functionalities (Scheffer and van Nes 2007; Yang et al. 2010). However, the rapid deterioration of these ecosystems presents a global challenge, as evidenced by the decline in both the abundance and diversity of aquatic plants (Davidson 2014; Zhang, Jeppesen et al. 2017). Research indicates that the eutrophication‐induced decline of submerged plants may trigger a transition from macrophyte to algal dominance, resulting in reduced biodiversity and degradation of habitats (Zhang, Jeppesen et al. 2017). As primary producers in lake ecosystems, community structures and diversities of aquatic plants are critical for sustaining ecosystem health and functions (Jeppesen et al. 1998; Scheffer 1998). Thus, investigating the composition and successional traits of aquatic plant communities is essential for comprehending the changes in lake ecosystems' structure and function, including their dynamics. Such focus is imperative for lake research and necessary for aquatic ecosystem restoration (Bennion et al. 2018).

The current understanding of long‐term changes in aquatic plant communities primarily relies on historical field surveys and analyses of satellite imagery (Huang et al. 2021; Luo et al. 2016; Wang et al. 2016). However, these data collection efforts often commence subsequent to the degradation or extinction of macrophyte communities, resulting in relatively short documentation periods, typically spanning less than half a century. Furthermore, remote sensing archives face challenges such as species ambiguities and limited spatiotemporal resolution, which inherently impact the quality of the data.

Paleolimnological records provide a unique opportunity to reconstruct the evolutionary trajectory of aquatic plants through the analysis of proxies such as aquatic pollen, diatoms, cladocerans, chironomids, biomarkers, and stable carbon isotopes (Dong, Kattel, and Jeppesen 2020; Ge, Zhang, and Yang 2018; Karst and Smol 2000; Yang et al. 2006; Zeng et al. 2024; Zhang, Su et al. 2017). However, the limited abundance and taxonomic uncertainty of aquatic pollen in sediment records pose challenges for accurately reflecting past vegetation dynamics. Similarly, these indicators—diatoms, cladocerans, chironomids, and biomarkers—provide preliminary insights into variations in the abundance of aquatic plants but are insufficient to fully elucidate changes in community composition.

Aquatic plant remains, known as plant macrofossils, are preserved in sediments that include seeds, fruits, leaves, bud scales, cuticles, and other durable components (Birks 2001). These macrofossils can help us understand past communities and track changes in lake vegetation over time (Mazzini et al. 2015; Brzozowski et al. 2021). Recently, there has been increased interest in using plant macrofossils to reconstruct historical aquatic macrophyte in lakes within the field of lake paleoecology. Such reconstructions have been useful for restoring aquatic ecosystems (Madgwick et al. 2011; Salgado et al. 2010; Sayer et al. 2010). For instance, Salgado et al. (2010) analyzed plant macrofossils from a sediment core to reveal the historical succession patterns of aquatic vegetation in Loch Leven (Scotland) over 150 years. Their findings highlighted the importance of considering a lake's natural baseline condition before human intervention in ecological restoration projects. Similarly, Madgwick et al. (2011) highlighted the significance of protecting and rehabilitating floating‐leaved and emergent plant communities by reconstructing two centuries' worth of aquatic vegetation based on plant macrofossil analysis conducted at Barton Broad—a shallow eutrophic lake located in eastern England. Moreover, plant macrofossil analysis serves as a crucial resource for investigating the impact of hydrological changes on aquatic vegetation (Mazzini et al. 2015), as well as studying the long‐term effects of climate change on submerged vegetation communities spanning decades or even centuries (Brzozowski et al. 2021). However, much research has focused on temperate regions while leaving subtropical shallow lakes relatively unexplored for plant macrofossil exploration.

The Yellow River (Huanghe River), China's second‐largest river, boasts a rich history marked by channel modifications and devastating floods in its lower reaches (Shu and Finlayson 1993). Historical records indicate that numerous lakes once dotted these regions, significantly affected by flooding, river migration, and human activities (Zhang 1987). Regrettably, most of these lakes have either vanished or experienced significant shrinkage over time (Zhang 1987). Among these, Nansi Lake stands out as a large, macrophyte‐dominated shallow floodplain lake, representing the largest among the freshwater lakes in North China, which is composed of four sub‐lakes Nanyang Lake, Dushan Lake, Zhaoyang Lake, and Weishan Lake. In addition to possessing the typical characteristics of shallow lakes, floodplain lakes are naturally dynamic ecosystems that respond to variations in river discharge and flooding both spatially and temporally. These factors influence the productivity composition of macrophyte biomass as well as species richness (Sousa, Thomaz, and Murphy 2011; Van Geest et al. 2005).

Nansi Lake plays a crucial role as a pivotal reservoir for the East Route of the South‐to‐North Water Diversion Project (SNWDP‐ER). Previous research indicates that this project has led to increased water recharge into the lake along with material input while also impacting hydrodynamic conditions, hydrological cycle processes, and habitats for aquatic organisms (Qu et al. 2020). However, the limited availability of monitoring data hampers our understanding regarding how vegetation responds to natural occurrences and human‐induced impacts within not only Nansi Lake but also other lakes located within the lower Yellow River Basin.

Weishan Lake, situated in the southernmost region among the four sub‐lakes, stands as the largest one with comparatively superior water quality compared to its counterparts. Our primary research site was chosen as Weishan Lake to evaluate changes in macrophyte communities over the past 160 years by analyzing plant macrofossil remains. We hypothesized that the composition and development of aquatic macrophytes in Weishan Lake during this time period have been influenced by both natural flood events triggered by the Yellow River and anthropogenic activities, including pollutant inputs, water conservancy projects, as well as climate change. Our objective was to determine how well these remains reflect macrophyte composition, understand succession patterns in Nansi Lake, identify factors influencing these communities, and suggest conservation measures for reinstating ecological equilibrium in Nansi Lake and similar lakes within the lower Yellow River Basin.

## 2 Materials and Methods

### 2.1 Study Site

**FIGURE 1:**
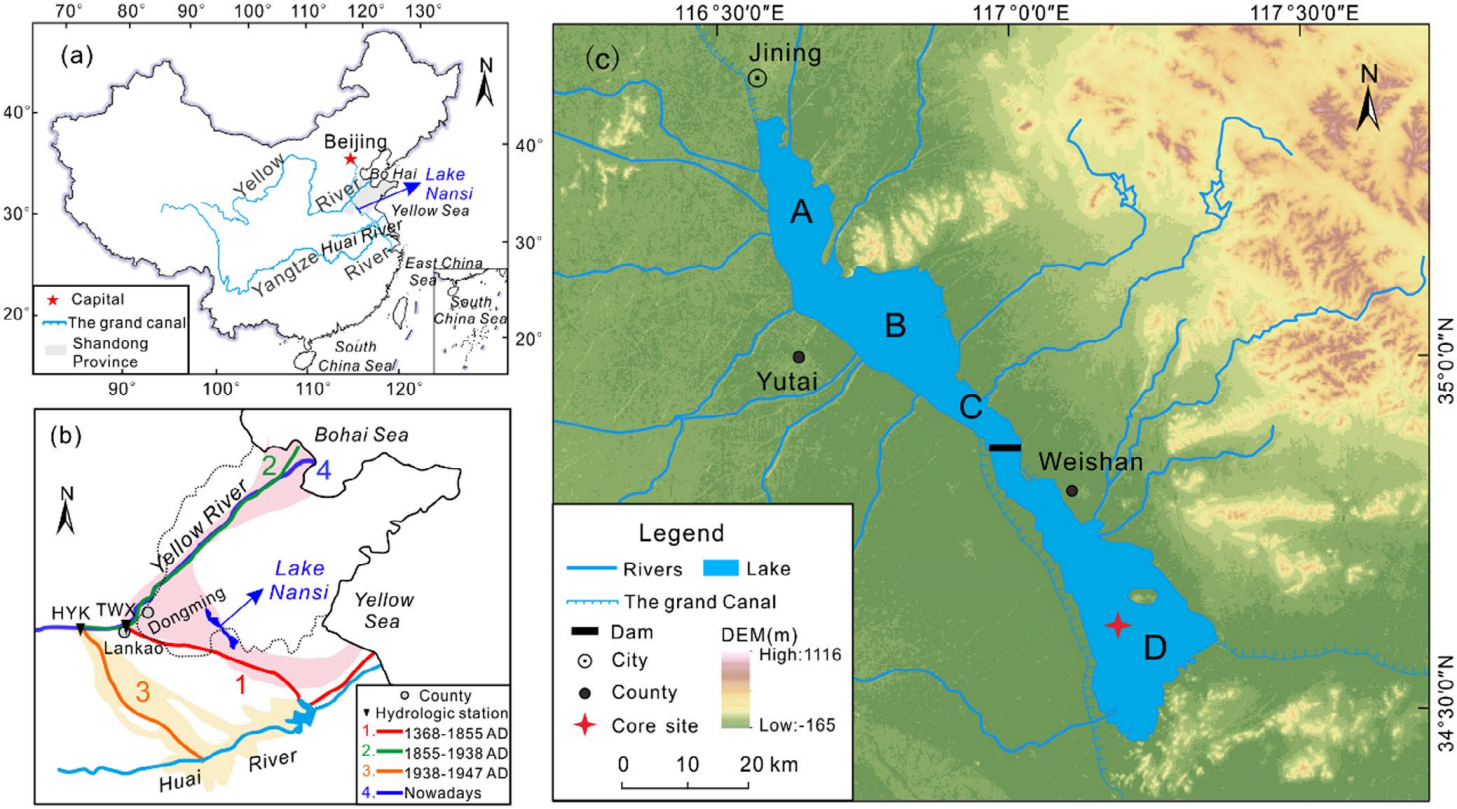
(a) Location of Nansi Lake in China and specifically in Shandong Province; (b) diversion of the lower Yellow River since 1386, the Yellow River flood area after 1855 (pink highlighted areas), and 1938 (orange highlighted areas); and (c) local setting, showing four sub‐lakes: Nanyang Lake (A), Dushan Lake (B), Shaoyang Lake (C), and Weishan Lake (D), with the core sampling site located in Weishan Lake.

Nansi Lake (34°27′–35°20' N, 116°34′–117°21′ E), located in the southwestern region of Shandong Province (Figure 1), is renowned as the largest freshwater lake in North China. It has an average depth of 1.46 m, covers an extensive water surface area of 1266 km^2^, and has a drainage area of 30,453 km^2^ (Wang and Dou 1998). Since the construction of a dam in 1960, the lake has been divided into upper and lower lakes.

The upper lakes encompass Nanyang Lake, Dushan Lake, and most parts of Zhaoyang Lake. These lakes receive inflows from a total of 29 rivers within a catchment area spanning over 26,934 km^2^, accounting for approximately 88.4% of the entire catchment area (Table 1). On the other hand, the lower lakes mainly consist of Weishan Lake with a small portion belonging to Zhaoyang Lake. They are fed by a network of 24 rivers; however, there is minimal contribution from large rivers to these lakes. The water collection area for the lower lakes covers about 3519 km^2^, representing around 11.6% of the overall collection area (Table 1). In terms of hydrological characteristics, there is a discrepancy in water levels between the upper and lower lakes. The eastern part of the Nansi Lake Basin exhibits a mountainous and hilly topography, while the western part is characterized by plains (Figure 1c). The terrain follows a north–south orientation with higher elevations predominantly located in the northern region (Figure 1c). All river systems within the drainage basin flow radially toward the lake area (Zhang et al. 2020).

**TABLE 1:** Hydraulic characteristics of Nansi Lake (Ding et al. 2015).

	The upper lakes	The lower lakes	Total
Surface area km^2^	602	664	1266
Catchment area km^2^	26,934	3519	30,453
Maximum storage capacity 10^8^ m^3^	24.5	25.7	50.2
Number of inflowing rivers	29	24	53
Number of outflowing rivers	0	4	4
Maximum depth m	2.76	2.38	2.76
Mean depth m	1.56	1.35	1.46

Weishan Lake (116°58′ E–117°21′ E, 34°27′ N–34°52′ N) is situated in the southernmost region among the four sub‐lakes and holds the distinction of being the largest, covering an approximate area of 660 km^2^. With an average water depth of around 2.5 m and superior water quality compared to the other three sub‐lakes (Xu et al. 2019), Weishan Lake serves as a habitat for diverse aquatic macrophytes, predominantly including *Potamogeton lucens* , *Myriophyllum spicatum* , and *Potamogeton crispus* . These macrophytes collectively occupy approximately 89.9% of the lake's surface area, making it a prime example of a shallow lake dominated by macrophytes (Cao et al. 2019; Xu 2020). According to a field investigation conducted in 2019 (Tong et al. 2024), the lake exhibits a total phosphorus (TP) concentration of 0.086 ± 0.014 mg/L and a total nitrogen level of 2.367 ± 0.523 mg/L, accompanied by relatively low chlorophyll‐a concentrations ranging from 7.075 ± 3.924 mg/m^3^. The alkalinity remains neutral with a pH value of 8.627 ± 0.548, while the dissolved oxygen (DO) content measures at 9.351 ± 1.164 mg/L and water hardness stands at 60.05 mg/L (CaO), respectively.

**FIGURE 2:**
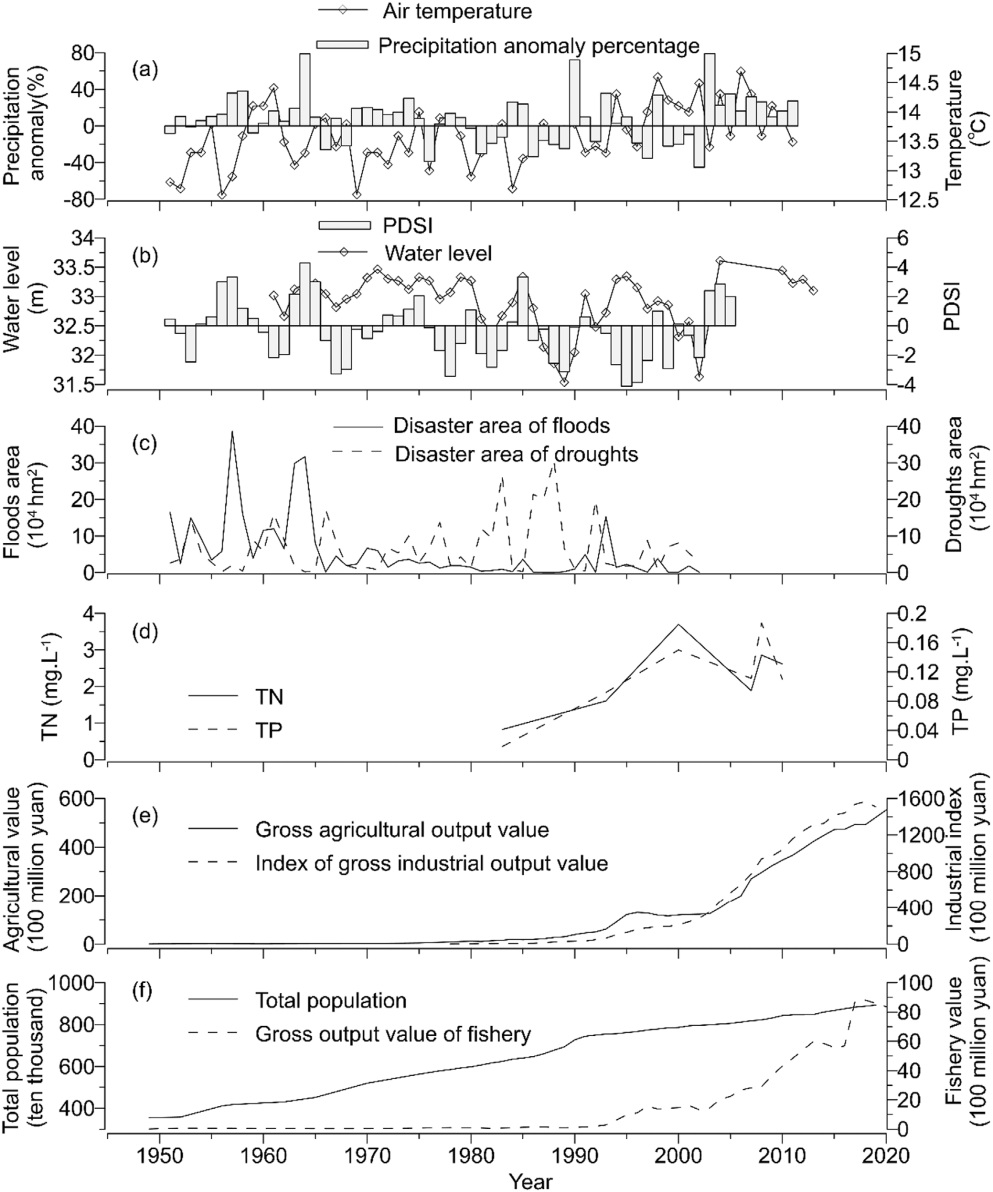
Documented records for Nansi Lake and its basin over the past approximately 70 years include: (a) mean annual temperature and annual precipitation anomaly percentage data from Yu et al. (2014); (b) water‐level fluctuations in Nansi Lake from Yang et al. (2009) and Wu et al. (2015), as well as Palmer Drought Severity Index within its basin; positive values represent moisture, while negative values indicate dryness; (c) information on the disaster area affected by floods and droughts from Zhang, Xin, and Liang (2007); (d) concentrations of total phosphorus (TP) and total nitrogen (TN) from Shu et al. (2012); and finally, data on agricultural and industrial output values (e), population count (f), and fisheries output (f) sourced from the China Statistical Yearbooks Database.

The region exhibits a semi‐humid warm temperate monsoon climate, characterized by an annual mean temperature of 14.2°C and average precipitation of 684 mm per year, accompanied by an annual mean evaporation rate of 704 mm (Wang and Dou 1998). Precipitation displays significant interannual variability, resulting in spring and autumn droughts as well as summer floods (Zhang et al. 2020). The Nansi Lake Basin frequently experiences both floods and droughts. Historically, there was a recurring cycle of droughts and floods, with floods being more common (Figure 2c). However, since the 1980s, exacerbated by arid conditions, water diversion upstream, and excessive water consumption near the lake area, drought severity has surpassed that of floods (Zhang, Xin, and Liang 2007). Nansi Lake is acknowledged as one of the primary freshwater fishery production bases in Shandong Province, boasting abundant fish resources. Following the adoption of natural fishing practices in the 1950s and 1960s, cage culturing was introduced in the 1990s, leading to extensive cultivation of crabs and fish within cages (Wang 2014).

### 2.2 Historical Aquatic Macrophyte and Environmental Data

To validate our paleoecological reconstruction of macrophytes, we obtained historical records of aquatic plants from published sources (Jining Science and Technology Commission 1987; Ke et al. 2018; Shi 2002). These records provided a comprehensive list of aquatic plants surveyed in several years for comparison with our paleoecological data. However, when discussing the representativeness of plant residues in this study, we focused only on submerged taxa with photosynthetic parts that predominantly grow underwater and excluded wetland and emergent plants (Davidson et al. 2005). This exclusion was justified as wetland and emergent plants tend to be deposited near their source plant close to the shorelines and are rarely found at core sites.

To gather background information on the sedimentary records analyzed in this study, we collected long‐term historical data from various sources including published studies (Shu et al. 2012; Wu et al. 2015; Yang et al. 2009; Yu et al. 2014; Zhang, Xin, and Liang 2007) and the China Statistical Yearbooks Database. These data included parameters such as climate, hydrology, and human activities for Lake Nansi and its surrounding basin (Figure 2a–f). Major transformations have been observed in Lake Nansi and its surrounding region over the past approximately 70 years due to the combined impacts of climate change and anthropogenic activities.

### 2.3 Sediment Sampling and Analyses

In October 2018, a 60 cm long sediment core was collected at a water depth of 2.2 m in Lake Weishan, using a gravity corer with an outer diameter of 60 mm (34°36′39″ N, 117°11′16″ E; Figure 1c). Sub‐samples were taken at intervals of 1 cm in the field and stored below temperatures of 4°C until further processing for analysis. The sub‐samples underwent examination for radionuclide activity (^210^Pb and ^137^Cs), particle size, loss on ignition (LOI_550_ and LOI_950_), and plant macrofossils.

The specific activity test of ^210^Pb and ^137^Cs involves a gamma‐energy spectrum analysis system consisting of an HPGe well detector (HPGe GwL‐120‐15, EG&G, USA) manufactured by EG&G ORTEC and a 16 K multichannel analyzer integrated with an IBM microcomputer. The specific activity of ^210^Pb is determined based on the gamma‐ray peak area at 46.5 keV. The short‐lived radionuclide ^214^Pb, derived from the decay of ^226^Ra, is used to calculate the specific activity of ^226^Ra through spectral peak area measurement (at 352 keV) (Appleby 2001; Nahm et al. 2010). The difference between the specific activities of ^210^Pb and its parent nuclide, i.e., ^226^Ra, provides us with the specific activity value for ^210^Pb_ex_. Similarly, the specific activity of ^137^Cs is calculated using the peak area in its gamma‐ray spectrum at 662 keV. Finally, the deposition age was determined by measuring these specific activities.

The Malvern Mastersizer‐2000 laser optical particle‐size analyzer (Malvern, United Kingdom) was used to measure the particle size distribution. Prior to analysis, carbonates were eliminated by treating with 10% HCl, and organic matter was removed using 30% H_2_O_2_ along with sodium hexametaphosphate as a dispersing agent. Clay particles were categorized as less than 4 μm, fine silt ranged from 4 to 16 μm, coarse silt spanned between 16 and 64 μm, and sand particles were larger than 64 μm. The median particle size (MD) was calculated to determine the central tendency of the particle size distributions.

The Loss on Ignition (LOI) was determined using standard procedures, involving a 5 h combustion at 550°C and a 2 h combustion at 950°C (Heiri, Lotter, and Lemcke 2001). The LOI method is commonly used to measure the mass loss of lacustrine sediments at specific temperatures (550°C and 950°C) in order to quantify the content of organic matter and carbonates. LOI_550_ represents the complete decomposition of organic carbon, while LOI_950_ indicates the complete decomposition of carbonates (Yang et al. 2015).

For processing plant macrofossil samples, we followed the standard procedure (Birks 2001): sediment samples of 25–50 cm^3^ were extracted and dispersed using a solution containing 10% Na_4_P_2_O_7_·10H_2_O as a dispersant. The dispersed sediment was then sieved through mesh sieves with pore sizes of 250 μm and 125 μm to obtain plant residues of different sizes. These residues were placed in a petri dish filled with water (depth: 2–3 mm) and identified and counted under a microscope (magnification: 10–100×). All residues larger than 250 μm were counted, while one‐fourth of the residues within the size range of 125–250 μm were counted and adjusted to represent the total count. The identification process primarily involved aquatic plant seeds, fruits, leaves, oospores (such as *Chara*), and scaly roots. Most specimens could be identified at the genus or species level using published model species plates literature by Beijerinck (1947), Berggren (1969, 1981), Birks (2001), Haas (1994), Mauquoy & van Geel (2007), the Scientific Database of Chinese Plant Species (http://db.kib.ac.cn/CNFlora/SearchEngine.aspx), and previously collected remnants of aquatic plants. Additionally, certain animal remains such as cladoceran ephippia and bryozoan statoblasts were also identified during the macrofossil analysis.

The TiliaGraph procedure was used to illustrate the temporal variation in plant macrofossil concentrations in the lake, generating a stratigraphic figure. These concentration levels directly reflect changes in diverse aquatic plants, providing valuable insights into the composition and evolution of aquatic plant communities over time. To analyze ecological succession patterns within these communities, all plant macrofossil species were categorized based on different types: submerged, floating‐leaved, free‐floating, and emergent plants. Additionally, variations in the diversity of aquatic plants during different time intervals were demonstrated using Hill's N2 diversity index (Hill 1973) combined with major environmental variables.

### 2.4 Data Analysis

The plant macrofossil zones in the core were identified using the constrained incremental sum of squares (CONISS) facility in computer programs TILIA and TILIAGRAPH (Grimm 2011). Temporal patterns of the macrophyte community and their relationships with invertebrate changes and environmental parameters were explored through multiple factor analysis (MFA) using R packages “FactoMiner” and “Factoextra” (Pagès 2002). An MFA was initially conducted to compare sediment core records from the past 160 years, including macrophytes, bryozoans, cladocerans, physical parameters, and geochemical parameters. This multivariate technique facilitated classifying core depths based on quantitative variable sets grouped into categories such as macrophytes, bryozoans, cladocerans, physical data (particle size), and geochemical data (LOI_550_ and LOI_950_). Subsequently, a narrower time period from the mid‐20th century to present day was examined to investigate the impact of natural and anthropogenic variables on long‐term macrophyte fluctuations in the lake. The MFA incorporated analyses of sediment core groups along with climatic factors (water level and disaster areas of floods and droughts), hydrological factors (water level, disaster areas of floods and droughts), and human‐related factors (total agricultural, industrial, and fishery output values, as well as total population). All data underwent ln (*x* + 1) transformations prior to MFA analysis for consistency across variables. This comprehensive analysis considered contributions from all variable groups when determining multivariate spatial distances between core depth samples (Salgado et al. 2020).

## 3 Results

### 3.1 Chronology

**FIGURE 3:**
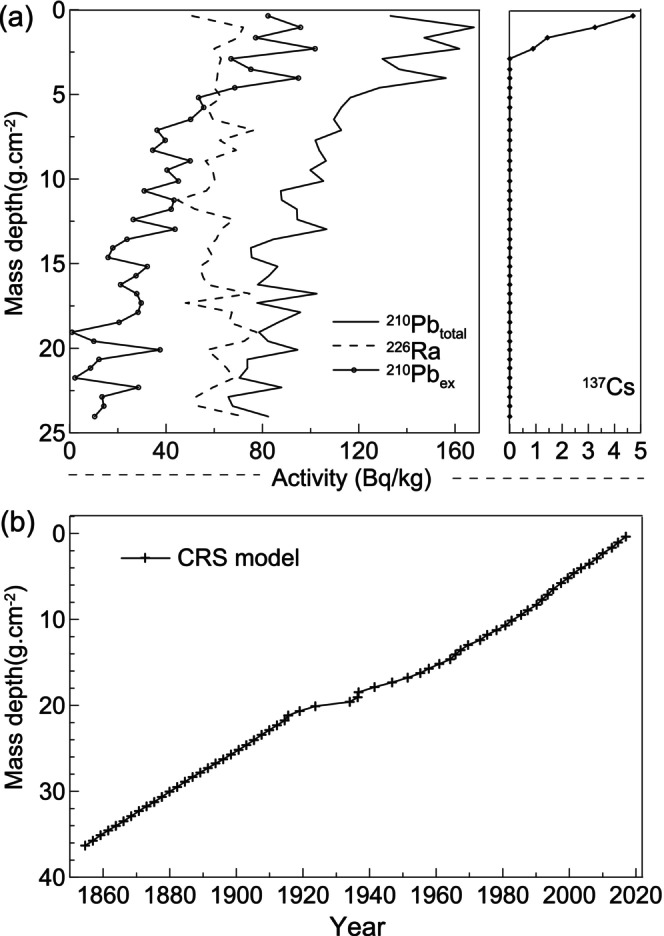
Chronology of the Weishan Lake core: (a) activity of ^210^Pb_total_, ^226^Ra, ^210^Pb_ex_, and ^137^Cs, (b) age–mass depth model. CRS: Constant rate of supply.

Considering sediment compaction, the mass depth of the sediment was used as a proxy for its actual thickness. The overall decline of ^210^Pb activity in the Lake Weishan core followed an exponential trend with increasing mass depth, although there were fluctuations within a wide range (Figure 3a). Therefore, we established an age–mass depth model for the Lake Weishan core based on the ^210^Pb constant rate of supply (CRS) model (Figure 3b). Dates below 22 g/cm^2^ were extrapolated using the average sedimentation rate, and the entire stratigraphy (36 g/cm^2^) represented sediments from approximately 1855–2018.

The activity of ^137^Cs in the Lake Weishan core shows a surface accumulation trend. In the sediment profile, the time when ^137^Cs was first detected (validated by the ^210^Pb chronology) is significantly delayed compared to its initial deposition in the early 1950s. This observation aligns with a previous study conducted in Lake Nansi, suggesting that substantial losses of ^137^Cs from sediment through diffusion and outflows may contribute to this phenomenon (Ding et al. 2015). Additionally, Lake Nansi's characteristics as a large shallow lake dominated by macrophytes, abundant aquatic plants, and organic matter concentrated primarily in the upper sediment layers derived from decaying remains within the lake ecosystem may play a role. Since ^137^Cs has a high affinity for adsorption onto organic matter, it accumulates toward the surface accordingly. Therefore, we conclude that using ^137^Cs as a temporal indicator for our research is impractical.

### 3.2 Plant Macrofossils

**FIGURE 4:**
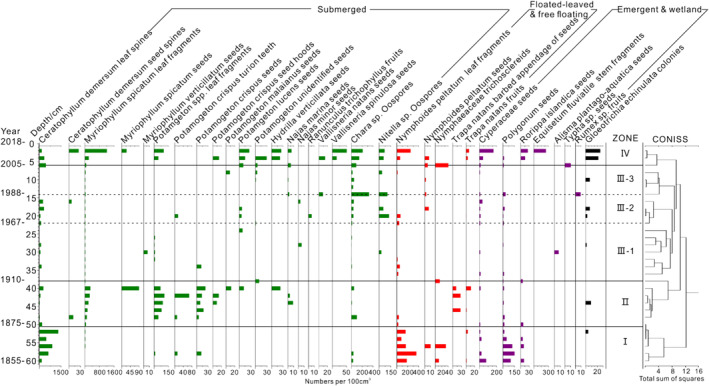
Stratigraphic plots of macrophyte remains (green—submerged, red—floated‐leaved/free floating, and purple—emerged/wetland) in the Weishan Lake core. Note the variable scaling of the *x*‐axis. Figures S1 and S2 showcase photographs of these remains.

The analysis of plant macrofossils identified a total of 26 taxa, including 15 submerged species (*Potamogeton lucens*, *Potamogeton malaianus, Potamogeton maackianus*, *Potamogeton crispus* , *Myriophyllum spicatum* , *Myriophyllum verticillatum* , *Ceratophyllum demersum* , *Hydrilla verticillata* , *Najas marina* , *Najas minor* , *Vallisneria natans* , *Vallisneria spinulosa*, *Chara* sp., *Nitella* sp., and *Ranunculus trichophyllu*), four floating‐leaved species ( *Trapa natans* , *Euryale ferox* , Nymphaeaceae, *Nymphoides peltatum*), and seven emergent/wetland species (Cyperaceae, *Polygonum* sp., *Typha* sp., *Rumex* sp., *Alisma plantago*, *Rorippa islandica* , and *Equisetum fluviatile* ). CONISS was used to divide the core sample into four zones and further subdivide them into three sub‐zones (Figure 4).

Zone I, the lowest zone (60–51 cm; ca. 1855–1875), exhibited a diverse assemblage of submerged *C. demersum* , floating *N. peltatum* , emergent/wetland taxa including *Polygonum* sp., Cyperaceae, and *R. islandica* , along with sporadic occurrences of *P. crispus* , *Chara* sp., and Nymphaeaceae. The average density per 100 cm^3^ was recorded at 1139 individuals. Additionally, there was a relatively high abundance of ephippia from littoral cladocerans and statoblasts from bryozoans.

Zone II (51–37 cm; ca. 1875–1910) witnessed a rapid increase in the concentrations of submerged macrofossils, with an average of 1999 individuals per 100 cm^3^. Remarkably, *P. crispus* and *M. spicatum* emerged as dominant species, while novel taxa such as *P. lucens* , *P. malaianus*, *H. verticillata* , and *N. marina* were observed. However, the abundance of *C. demersum* remains declined. Floating‐leaved aquatics showed a significant decrease, with *N. peltatum* experiencing a pronounced decline and *T. natans* experiencing an exponential growth. Emergent species had sporadic occurrences with an overall reduction in concentration.

In Zone III (37–6 cm; ca. 1910–2005), there was a significant decline in the concentrations of submerged, floating, and emergent plants, with an average concentration of 302 individuals per 100 cm^3^. Episodic occurrences of *C. demersum* , *Potamogeton* species, *M. verticillatum* , *N. minor* , Charophyta, and *N. peltatum* were noted but at low concentrations. Bryozoans and littoral cladocerans also showed reduced abundance. Zone III was divided into three subzones: III‐1 (37–22 cm; ca. 1910–1967), III‐2 (22–14 cm; ca. 1967–1988), and III‐3 (14–6 cm; ca. 1988–2005). In Zone III‐1, most species either disappeared or experienced a substantial decline in abundance compared to Zone II. However, within Zone III‐2, there was an increase in the concentration of remains for specific submerged plants like Charophyta (*Chara* and *Nitella*), accompanied by a significant enhancement in the N2 diversity index. Nevertheless, starting from Zone III‐3 onward, both macrofossil remains' concentrations and the N2 diversity index declined subsequently.

In Zone IV (6–0 cm; ca. 2005–2018), macrofossil concentrations significantly increased, with most species either appearing or experiencing abundant growth. Submerged species reached their highest values throughout the core, with an average concentration of 5855 individuals per 100 cm^3^ of the sediment. Dominant submerged species included *M. spicatum* , *P. lucens* , *H. verticillata* , *C. demersum* , *Vallisneria* species, and Charophyta. Additionally, there was a noticeable increase in floating‐leaved aquatic remains primarily represented by *N. peltatum* , Nymphaeaceae, and *T. natans* . Furthermore, emergent/wetland taxa showed a significant rise with an average concentration of 195 individuals per 100 cm^3^ including Cyperaceae, *R. islandica*, and *E. fluviatile* .

### 3.3 Comparison of the Historical and Macrofossil Records

In total, there were 35 aquatic taxa documented across the three surveys in the historical records: 12 submerged plants, 13 floating‐leaved plants, and 10 floating plants (Table 2). A successful identification was achieved for 19 species, representing 54.3%, based on the analysis of plant macrofossils. This *includes* 15 submerged and 4 floating‐leaved taxa (see Table 2). Prominent historical macrophyte species, such as *H. verticillata* , *M. spicatum* , *P. lucens* , *P. crispus* , *C. demersum*, and *N. peltatum* , exhibited a significant presence based on their well‐preserved macrofossils. Plant macrofossils uncovered five previously unrecorded submerged taxa including *M. verticillatum* , *R. trichophyllus* , *Chara* sp., *Nitella* sp., and *V. spinulosa* . However, two specific submerged taxa, *Utricularia vulgaris* and *Potamogeton pectinatus* , lacked discernible sedimentary remains. Hence, the plant macrofossils effectively captured the predominant components of the aquatic flora in Weishan Lake with a particular emphasis on representing submerged plants' diversity. However, limited representation in fossilized remains restricts documentation to only four taxa among numerous floating‐leaved and free‐floating plants (Nymphaeaceae, *Trapa* sp., and *N. peltatum* ).

**TABLE 2:** Aquatic macrophytes recorded in Nansi Lake 1983, 1995–1996, and 2010 with information on their representation by macrofossils (M) in the sediment core. (*shows modern plants in the survey records; M shows macrofossils found in the sediment core; − shows no record;? shows Trapa remains can only be identified as genera and not as specific species.)

Vegetation type	Species	1983	1995–1996	2010	Macrofossils
Submerged taxa	*Ceratophyllum demersum*	*	*	*	M
	*Myriophyllum spicatum*	*	*	*	M
	*Myriophyllum verticillatum*	—	—	—	M
	*Utricularia vulgaris*	*	*	—	—
	*Potamogeton crispus*	*	*	*	M
	*Potamogeton malaianus*	*	*	*	M
	*Potamogeton lucens*	*	*	*	M
	*Potamogeton maackianus*	*	*	*	M
	*Potamogeton pectinatus*	*	*	*	—
	*Najas marina*	*	*	—	M
	*Najas minor*	*	*	—	M
	*Hydrilla verticillata*	*	*	*	M
	*Vallisneria spiralis/natans*	*	*	*	M
	*Vallisneria spinulosa*	—	—	—	M
	*Nitella* sp.	—	—	—	M
	*Chara* sp.	—	—	—	M
	*Ranunculus trichophyllus*	—	—	—	M
	Total submerged	12	12	9	15
Floated‐leaved taxa	*Potamogeton distinctus*	*	*	—	—
	*Marsilea quadrifolia*	*	*	—	—
	*Polygonum amphibium*	*	—	—	—
	*Nelumbo nucifera*	*	*	—	M
	*Euryale ferox*	*	*	—	M
	*Trapa quadrispinosa*	*	—	*	?
	*Trapa acornis*	*	*	—	?
	*Trapa pseudoincisa*	*	*	—	?
	*Trapa potaninii*	*	—	—	?
	*Trapa bispinosa*	*	—	—	?
	*Nymphoides peltatum*	*	*	*	M
	*Nymphoides indica*	*	*	—	—
	*Trapella sinensis*	*	*	—	—
	Total floated‐leaved	13	9	2	4
Free‐floating taxa	*Hydrocharis dubia*	*	*	*	—
	*Ceratopteris pteridoides*	*	*	—	—
	*Azola imbricata*	*	*	—	—
	*Salvinia natans*	*	*	*	—
	*Lemna trisulca*	*	*	—	—
	*Lemna minor*	*	*	*	—
	*Spirodela polyrrhiza*	*	*	—	—
	*Wolffia arrhiza*	*	—	—	—
	*Eichhornia crassipes*	—	*	*	—
	*Pistia stratiotes*	—	*	—	—
	Total free‐floating	8	9	4	0
Total aquatic taxa		33	30	15	19

### 3.4 Multiple Factor Analysis

**FIGURE 5:**
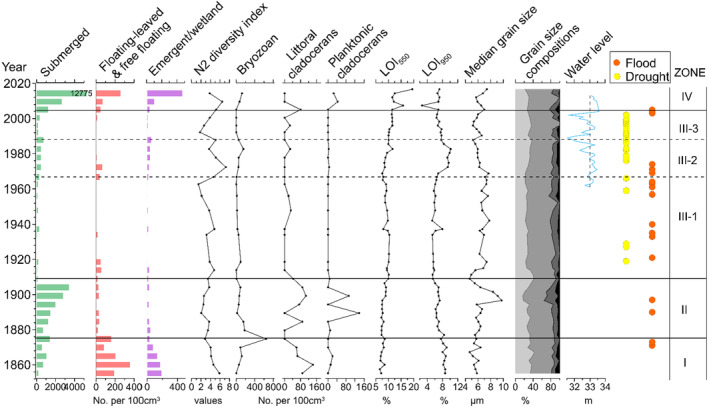
Long‐term records collected for Weishan Lake, including macrofossil density of submerged, floating‐leaved and free‐floating and emergent/wetland plants; macrofossil N2 diversity index; bryozoan statoblast density; littoral and planktonic cladocerans ephippia density; LOI_550_ and LOI_950_ measurements; median grain size and grain size compositions (from left to right: clay, fine silt, coarse silt, and sand); and water‐level fluctuations and flood/drought records.

The long‐term ecological and environmental evolution is compared comprehensively using various parameters, including plant macrofossils (submerged, floating‐leaved and free‐floating, emergent and wetland plants, and the N2 diversity index of macrophytes), animal remains (such as bryozoan statoblast density and littoral and planktonic cladoceran ephippia), physicochemical data (LOI_550_, LOI_950_, and grain size compositions/median grain size), and hydrological data (flood and drought records, water level). Figure 5 presents a comprehensive comparison of these parameters.

**FIGURE 6:**
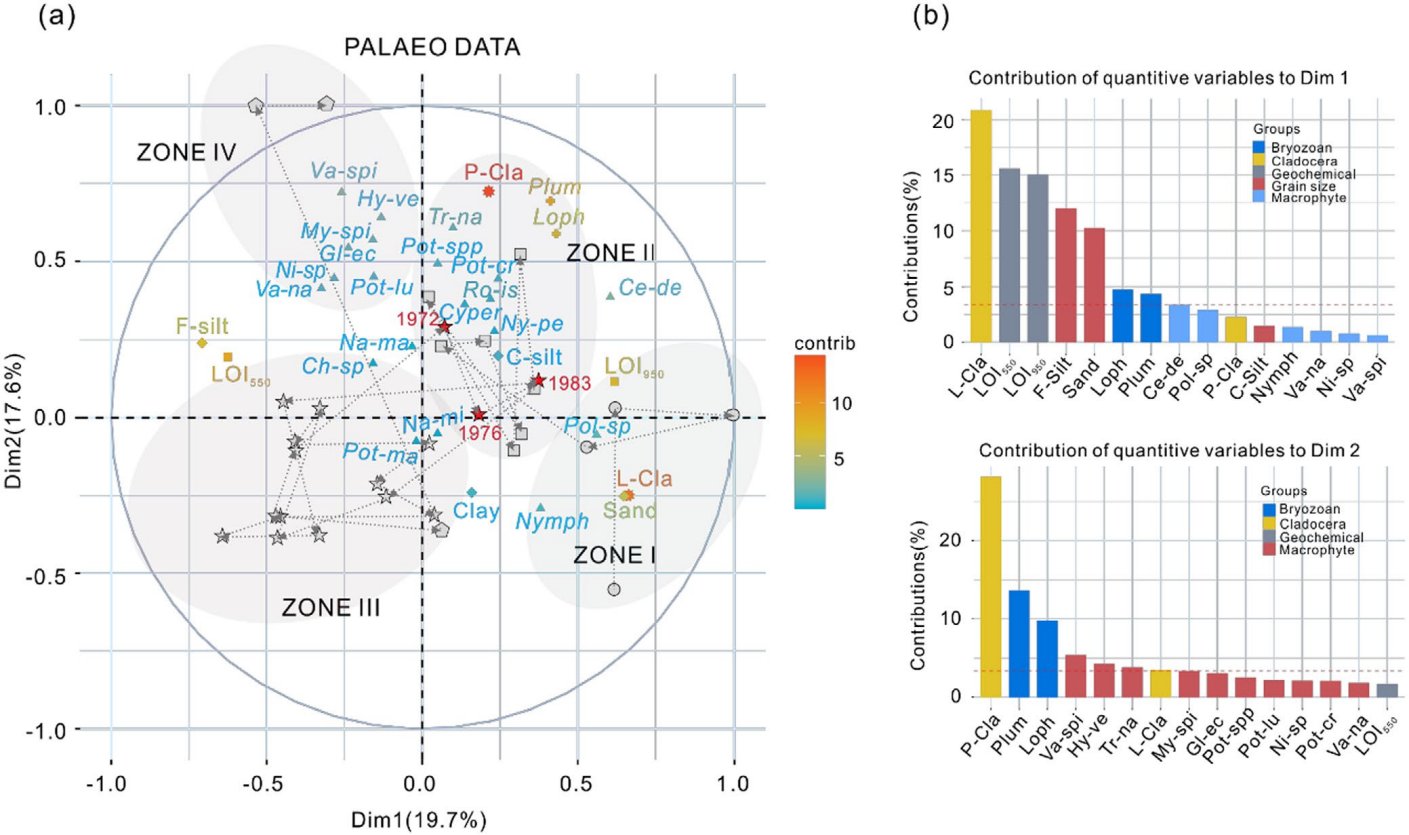
Multiple factor analysis (MFA) plots for the entire sediment core records, showing (a) variation and contribution of biological (macrophyte—triangles; sessile invertebrates *Plumatella* and *Lophopodella*—crosses; Cladocera—star, geochemical (LOI_550_ and LOI_950_‐squares) and physical (grain size—rhombuses) sedimentary data for the entire Weishan Lake core. Major zones of macrophyte community changes are indicated with circles (Zone I, light purple area), stars (Zone II, light orange area), squares (Zone III, light blue area), and pentagons (Zone IV, light pink area). The contribution of each variable is indicated according to a color scale, with red denoting the highest and green the lowest value. Black dashed arrows represent the temporal trajectory of the sediment sample change. (b) contributions of quantitative variables to dimension 1 and dimension 2. *My‐spi* = *Myriophyllum spicatum* , *Pot*‐spp = *Potamogeton* spp., *Tr‐na* = *Trapa natans* , *Va‐spi* = *Vallisneria spinulosa*, *Hy‐ve* = *Hydrilla verticillata* , *Pot‐cr* = *Potamogeton crispus* , *Gl‐ec = Gloeotrichia echinulate, Pot‐lu = Potamogeton lucens, Cyper* = Cyperaceae, *Ro‐is* = *Rorippa islandica* , *Na‐ma* = *Najas marina* , *Ch*‐sp = *Chara* sp., *Ni*‐sp = *Nitella* sp., *Ny‐pe* = *Nymphoides peltatum*, *Ce‐de* = *Ceratophyllum demersum* , *Va‐na* = *Vallisneria natans* , *Pot‐ma* = *Potamogeton malaianus, Na‐mi* = *Najas minor* , *Nymph* = Nymphaeaceae, *Pol‐sp* = *Polygonum* sp., *Plum* = *Plumatella*, *Loph* = *Lophopodella*, L‐Cla = littoral cladocerans, P‐Cla = planktonic cladocerans, F‐silt = fine silt, C‐silt = coarse silt.

The MFA analysis conducted on the complete sediment core records revealed the first two dimensions accounted for 37.3% of the total variation, effectively distinguishing the four macrophyte zones in the MFA biplot (Figure 6). Samples from Zones I and II vary along the gradient of high sand and organic content, mainly consisting of emergent *Polygonum* sp., floating‐leaved Nymphaeaceae, alongside littoral Cladocera, moving toward the gradient of coarser silt content and increased concentrations of submerged macrophyte remains, bryozoan genera *Lophopodella* and *Plumatella*, and planktonic cladocerans (Figure 6). In Zone III, there is a noticeable reduction in the abundance of both macrophyte and animal remains; however, it is worth noting that samples taken at a depth of 16–20 cm (approximately corresponding to the years 1972–1983) were found adjacent to Zone II. Finally, Zone IV displays heightened concentrations of various types of macrophytes, suggesting a recent shift into a new state for the lake.

**FIGURE 7:**
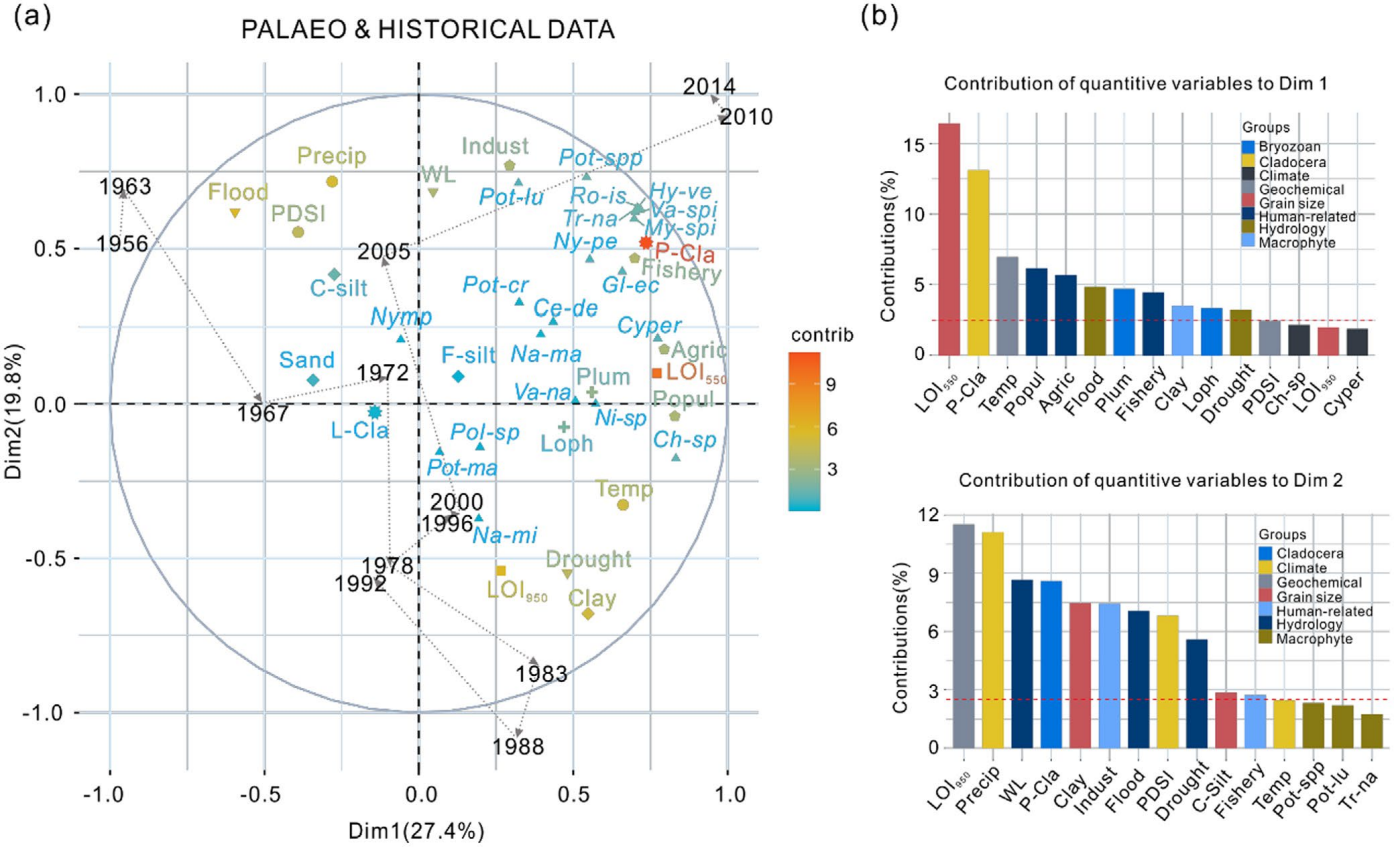
Multiple factor analysis (MFA) plots for the period 1956–2018 for which historical data were available, showing (a) variation and contribution of the biological, physical, and geochemical sedimentary data with historical hydrological data (WL, Flood and Drought—inverted triangles), climatic data (Temp, Precip and PDSI—circles), human data (Popul, Indust, Agric and Fishery—pentagons). The contribution of each variable is indicated according to a color scale, with red denoting the highest and green the lowest value. Black dashed arrows represent the temporal trajectory of the sediment sample change. (b) Contributions of quantitative variable groups to dimension 1 and dimension 2. *My‐spi* = *Myriophyllum spicatum* , *Pot*‐spp = *Potamogeton* spp., *Tr‐na* = *Trapa natans* , *Va‐spi* = *Vallisneria spinulosa*, *Hy‐ve* = *Hydrilla verticillata* , *Pot‐cr* = *Potamogeton crispus* , *Gl‐ec = Gloeotrichia echinulate, Pot‐lu = Potamogeton lucens, Cyper* = Cyperaceae, *Ro‐is* = *Rorippa islandica* , *Na‐ma* = *Najas marina* , *Ch*‐sp = *Chara* sp., *Ni*‐sp = *Nitella* sp., *Ny‐pe* = *Nymphoides peltatum*, *Ce‐de* = *Ceratophyllum demersum* , *Va‐na* = *Vallisneria natans* , *Pot‐ma* = *Potamogeton malaianus, Na‐mi* = *Najas minor* , *Nymp* = Nymphaeaceae, *Pol‐sp* = *Polygonum* sp., *Ty*‐sp = *Typha* sp., *Plum* = *Plumatella*, *Loph* = *Lophopodella*, L‐Cla = littoral cladocerans, P‐Cla = planktonic cladocerans, F‐silt = fine silt, C‐silt = coarse silt, Precip = precipitation, WL = water level, PDSI = palmer drought severity index, Flood = disaster area affected by floods, Drought = disaster area affected by droughts, Temp = air temperature, Popul = populations, Agric = agricultural output values, Indust = industrial output values, Fishery = fisheries output values.

During the period 1956–2014, an MFA of sediment records, historical climatic and hydrological data, as well as social parameters, revealed that the first two dimensions accounted for 47.2% of the total variation (Figure 7). The temporal trajectory of sediment sample changes during this period suggests that the lake has undergone intricate and profound alterations in its aquatic macrophyte composition over the past six decades (Figure 7).

## 4 Discussion

### 4.1 Long‐Term Dynamics of Aquatic Macrophytes

Our findings demonstrate that the dominant macrophyte species historically documented in Weishan Lake, such as *H. verticillata* , *M. spicatum* , *P. lucens* , *P. crispus* , *C. demersum,* and *N. peltatum* , are well represented by plant macrofossils. These macrofossils constitute approximately 54.3% of the documented aquatic species in Nansi Lake. This discovery surpasses previous comparative studies conducted on other shallow lakes in Europe (Davidson et al. 2005; Madgwick et al. 2011; Salgado et al. 2010; Kornijów et al. 2016; Kowalewski et al. 2013) and China's Yangtze region (Zhang et al. 2019, 2022), which reported preserved macrophyte proportions ranging from 37% to 49% using plant macrofossil records from sediment cores. Our result records date back to before the formation of unified Nansi Lake and extend until present times, encompassing the period following the implementation of SNWDP‐ER. These records offer a comprehensive historical account of how aquatic macrophytes have responded to both natural changes and human activities over time periods spanning climate variations and anthropogenic influences alike. Through multiproxy analysis and MFA (as depicted in Figures 5, 6, 7), we elucidate the impacts of hydrological changes and pollution input on the composition and succession patterns of macrophyte communities in Weishan Lake throughout its history, driven by flood disturbances, climate variations, and human actions. After analyzing multiple indices, long‐term watershed records, and MFA results, four key stages defining the evolution of aquatic macrophyte communities in Weishan Lake over a span exceeding 160 years were identified.

#### 4.1.1 Macrophyte Communities Before the Formation of the Unified Nansi Lake (Zone I; Pre‐ca. 1875)

From 1855 to 1875, the macrophyte community in Lake Nansi was dominated by emergent and wetland plants such as *Polygonum* sp., *R. islandica* , and Cyperaceae species. Additionally, floating‐leaved species like *N. peltatum* and Nymphaeaceae species were present, along with an underwater rootless submerged taxa *C. demersum* . These findings suggest that the lake exhibited swamp‐like environmental conditions during this period due to the preference of these plants for stagnant or slow‐moving waters (Klok and van der Velde 2022; Syed et al. 2018). The presence of abundant littoral cladocerans associated with macrophytes and detritus further supports a littoral environment (Salgado et al. 2020). Documentary evidence indicates shrinkage and declining water levels in the lake during that time due to diversion of Yellow River (Yu et al. 2004), which further confirms this observation.

Historically, the Lake Nansi area has been significantly affected by Yellow River flooding due to its previous course along Route 1 (Figure 1b), which flowed into the Huai River before reaching the Yellow Sea prior to 1855. However, in 1855, due to avulsion and diversion downstream in Tongwaxiang, Lankao County, Henan Province (Figure 1b; route 2), the Yellow River changed its course and started discharging into the Bohai Sea instead. As a result of this change in the river flow pattern, reduced vulnerability to floods from the Yellow River was observed in the Nansi Lake area (Zhang et al. 2002), while at the same time, there was a decrease in lake size and water levels (Yu et al. 2004). An elevated LOI_950_ value indicating carbonate enrichment also reflects this decline in lake level (Li et al. 2019). The variability in median particle size and component content within Lake Weishan core sediments during this period is relatively minimal, suggesting a stable sedimentary environment at that time. Therefore, this phase was characterized by swampy‐like conditions with abundant emergent/wetland vegetation but limited submerged species.

#### 4.1.2 Flourishing Period of Submerged Plants Following the Integration of Lake Nansi (Zone II; ca. 1875–1910)

Between 1875 and 1910, significant changes occurred in the lake ecosystem as a result of the Yellow River avulsion. The breach of the dike in Dongming County, Shandong Province (see Figure 1b), led to the inundation and merging of Lake Nanyang, Lake Shaoyang, Lake Dushan, and Lake Weishan into a unified natural system now known as Lake Nansi. Consequently, water levels rose and transformed previously swampy areas into a larger body of water. The decrease in the carbonate content observed after the 1870s indicated the dilution of lake water caused by rising levels, supported further by abundant ephippia of planktonic cladocerans during that period, suggesting a well‐developed pelagic community sustained by high lake levels (Zhu, Wang, and Brancelj 2005). Except for a few years, variability in median particle size and component content within Lake Weishan's sediment core is minimal, reflecting a relatively stable sedimentary environment. A peak at 43 cm in median particle size dated approximately to 1897 likely indicates an influx of coarse particulate matter resulting from a significant flood event. This aligns with historical records documenting a flood occurrence in 1897.

These alterations significantly impacted the macrophyte community in Lake Weishan, leading to pronounced changes in submerged plant growth as evidenced by an increased abundance of their remains in lake sediments. This was accompanied by an increase in littoral cladocerans associated with macrophytes and detritus, indicating facilitated macrophyte growth within the lake at that time. Substantial modifications occurred in the composition of submerged macrophytes, including a marked increase in *Potamogeton* species (e.g., *P. crispus* , *P. malaianus*, and *P. lucens* ), *M. spicatum* , *H. verticillata* , and *N. marina* , while there was a decline in nonrooted *C. demersum* . Furthermore, there has been a noticeable degradation of emergent and floating plants due to reduced concentration of their remains caused by elevated water levels following habitat condition changes in the lake; this may have resulted in decreased germination and survival rates for emergent and floating plants or their movement toward the lake's edge as water levels rise. Therefore, it can be concluded that between 1875 and 1910 Lake Weishan maintained relatively stable hydrodynamic conditions with higher water levels and increased abundance of submerged macrophytes.

#### 4.1.3 Mass Disappearance of Macrophytes: Impacts of Floods, Droughts, and Human Activity (Zone III; ca. 1910–2005)

Between approximately 1910 and 1965, there was a significant decline in aquatic vegetation characterized by sporadic species distribution, including *C. demersum* , *P. lucens* , *P. crispus* , *M. spicatum* , *N. peltatum* , and *Euryale ferox* . The reduction in bryozoan statoblasts and littoral cladocerans further suggests diminished substrate attachment due to frequent droughts and floods during this period that altered water levels and hydrodynamic conditions. Floods can have a catastrophic impact on aquatic macrophyte communities due to high water levels and increased turbidity, which reduce light availability during the growing season (Coops, Beklioglu, and Crisman 2003). Conversely, drought events with low water levels can also harm plants through wave action in winter and desiccation in summer (Coops, Beklioglu, and Crisman 2003). Furthermore, flood events likely played a predominant role in shaping the evolutionary trajectory of aquatic macrophytes during the period 1910–1970 (Zone III‐1), while drought events gained dominance thereafter (Zone III‐2 and III‐3), as supported by the MFA results shown in Figure 7. Historical data indicate that since the 1970s, there has been a decrease in precipitation and frequent occurrences of droughts in the Nansi Lake basin, resulting in a continuous decline in water levels depicted in Figure 2.

However, in the MFA analysis, samples at a depth of 16–20 cm (around 1972–1983) were found adjacent to Zone II samples. These anomalous data points may suggest stable hydrology and declining water levels during the onset of drought, potentially facilitating abundant charophyte (*Chara* and *Nitella*) oospores and species recovery. Previous studies have demonstrated that charophytes adapt their reproductive behavior to cope with fluctuating water levels (Wang, Liu, and Yu 2015). For example, reduced water levels and increased light intensity can stimulate sexual reproduction and oospore maturation in charophytes (Casanova 1994; Casanova and Brock 1999; Wang and Yu 2008). Furthermore, contemporary studies indicate that both the number (Asaeda, Rajapakse, and Sanderson 2007; Brzozowski and Pełechaty 2020) and size of oospores (Brzozowski et al. 2019) can vary along a depth gradient, indicating that charophyte oospores may not only serve as sensitive bioindicators of water trophicity (Schubert et al. 2018; Brzozowski and Pełechaty 2022), but also reflect water level fluctuations (Brzozowski et al. 2019). This finding is consistent with previous research conducted at Lake Liangzi (Zhang et al. 2019) and Eastern Lake Taihu (Zhang et al. 2022) within the Yangtze River floodplain, which revealed a significant increase in charophytes when water levels were lowered and hydrological disturbances were minimized. Therefore, the sample at a depth of 16 cm exhibited a notable rise in charophyte oospores due to the profound desiccation event that occurred in Lake Nansi during 1989. Additionally, charophytes are wintering species well‐suited for growth under stable conditions characterized by clear water with low to moderate nutrient content. Thus, the abundant presence of charophytes at the beginning of Zone III‐2 indicates a stable aquatic environment with good quality. Previous research has indicated that until the mid‐1980s, Lake Nansi's water quality largely complied with drinking standards (Class III).

However, since the late 1980s, Lake Nansi has experienced significant fluctuations in water levels due to anthropogenic upstream water diversion and excessive evaporation caused by dry climatic conditions. Furthermore, untreated discharge of industrial and agricultural sewage into the Lake Nansi basin has resulted in the degradation of water quality (Tian et al. 2017), thereby impeding macrophyte proliferation. Consequently, there have been noticeable declines observed in both species diversity and abundance as indicated by the macrofossil record. Charophytes exhibited a distinct decline, while other species showed either a decline or fell below detection limits. By 2000, annual phosphorus input into the lake exceeded 382.1 tons, surpassing the national Class V water quality standard for phosphorus concentration in Lake Nansi's waters (Liu et al. 2008). Therefore, flood events were predominant before the 1980s; however, from the 1980s onward, drought and water pollution became more prevalent factors shaping the evolution of aquatic macrophytes.

#### 4.1.4 Macrophyte Alterations Following Ecological Restoration and the Implementation of Water Diversion Projects (Zone IV; After 2005)

Following the emergency ecological water transfers in December 2002, there was a sudden surge in lake water levels (Figure 2b). The increase in planktonic Cladocera also indicates the establishment of a well‐supported pelagic community of Cladocera due to the elevated water level during this phase. Furthermore, the implementation of pollution control measures in Nansi Lake was made possible by the construction of SNWDP‐ER project in 2002. Through rigorous environmental management and ecological restoration efforts, consistent improvement has been observed in the water quality within the Nansi Lake basin (Tian et al. 2017). Hydrological and water quality changes have significantly influenced the aquatic plant community. The plant macrofossil record demonstrates a remarkable increase both in concentration and species abundance of aquatic plant residues, reflecting thriving aquatic vegetation and high biodiversity during this period. Dominant assemblages include *P. lucens* , *M. spicatum* , *C. demersum* , *H. verticillata* , *V. spinulosa* , *N. peltatum* characterizing the aquatic macrophyte community. Additionally, an increase in charophyte species suggests improved water quality as they prefer stable oligotrophic waters with low to medium nutrient levels.

However, the implementation of the water diversion project in December 2013 has been found to have significant impacts on material input, hydrodynamic conditions, hydrological cycle processes, and habitat environments for aquatic organisms (Tan et al. 2022). For example, there was a notable increase in total phosphorus levels, ammonia nitrogen concentrations, chlorophyll‐a content, and algae density during the period from 2013 to 2015 coinciding with the initial operation of the SNWDP‐ER (Tan et al. 2022). These impacts could potentially affect both the structure and distribution of the macrophyte community within the lake. Our findings demonstrate a substantial rise in pollutant‐resistant species abundance (e.g., *M. spicatum* , *N. peltatum* ) within surface samples, indicating that an unprecedented transition has recently occurred in the lake, as depicted in MFA plots (Figures 6 and 7). Furthermore, our results highlight interspecific variation nuances observed through a gradual shift from *V. natans* dominance toward *V. spinulosa* prevalence in surface samples. This discrepancy may stem from the fact that *V. spinulosa* displays superior growth performance under elevated nutrient concentrations, while *V. natans* exhibits enhanced adaptability to low‐to‐moderate nutrient levels. These results align with our previous investigations conducted in Yangtze floodplain and Lake Dongping within Yellow floodplain which revealed a pronounced increase in *V. spinulosa* at the expense of *V. natans* when nutrient loading escalated. In summary, this period witnessed improvements in aquatic environment facilitating abundant vegetation growth and enhanced primary productivity within the lake; however, simultaneous environmental threats also exist.

### 4.2 Implications for Lake Management

Our study provides a comprehensive and uninterrupted record of the macrophyte communities in Weishan Lake before disturbance, thereby addressing crucial knowledge gaps in the ecological understanding of lakes in the lower Yellow River region. This research establishes a robust scientific foundation for developing effective restoration and management strategies.

Furthermore, plant macrofossil analysis revealed five previously undocumented submerged taxa: *M. verticillatum* , *R. trichophyllus* , *Chara* sp., *Nitella* sp., and *V. spinulosa* . Field investigations of aquatic vegetation can occasionally overlook rare plants and subtle interspecific variations due to factors such as dense vegetation, topographical constraints, fishing net barriers, or sampling methods. However, these overlooked species can yield valuable insights into environmental changes as they often leave substantial residues in sediments. For instance, charophyte species identified in this study frequently go unnoticed during field investigations but are recognized for their sensitivity to environmental factors. Monitoring their temporal changes can aid in assessing the ecological status during specific periods. Another challenge arises from *Vallisneria* species in the field due to their small reproductive organs, similar vegetative organs, and pronounced variability (Fu et al. 2019). Nevertheless, sedimentary macrofossil analysis enables differentiation between distinct species based on seed remains, thus identifying both *V. spinulosa* and *V. natans* in this study. Survey records may not fully represent the aquatic flora at a specific location; however, examining sedimentary macrofossils can overcome this limitation.

In accordance with the requirements of the SNWDP, comprehensive measures have been implemented in the Nansi Lake basin for water quality monitoring and pollution control. However, persistent challenges remain in restoring and protecting not only Lake Nansi but also other impounded lakes along the SNWDP, including pollutant inputs, introduction of invasive species, hydrological changes due to water diversion, and effects of climate change. To effectively address these issues, we suggest integrating paleoecological methods into standard ecological monitoring protocols used for evaluating water ecological quality. This integration could serve as an early warning system for lake managers and facilitate a scientific assessment of the progressive impacts of water environment restoration efforts. For example, incorporating an examination of biological indicators such as aquatic macrophytes through surface sediment analysis in routine assessments enables prompt evaluation.

## 5 Conclusions

This study highlights the potential of using plant macrofossils to reconstruct long‐term variations in macrophyte community components, abundance, and ecosystem health assessment when monitoring records are limited in the lower Yellow River region. Our findings unveil that Weishan Lake has undergone four significant shifts in its macrophyte community over the past 160 years. These changes have been influenced by the migration of the Yellow River, modifications in hydrology resulting from both climatic fluctuations and human activities (such as anthropogenic upstream water diversion and the implementation of the SNWDP‐ER), as well as alterations in water quality.

Our study demonstrated a good representation of plant macrofossils, accounting for 54.3%, particularly submerged plants. However, there is still scope for improvement in future investigations including collecting additional sediment core samples dedicated to plant macrofossil analysis and implementing cross‐sectional sampling across various sub‐lakes spanning from coastal zones to open water areas. These measures aim to optimize the extraction of valuable information about aquatic plant residues within lakes. Significant variations exist in water quality and biological communities among different lake regions, especially those with large areas and complex shorelines where the diversity of aquatic vegetation distribution tends to be higher. Additionally, insufficient sample size during the post‐water transfer period resulted in inadequate discussion regarding the response of aquatic plant communities to water transfer projects; this aspect will receive more attention in subsequent studies.

## Author Contributions

**Qinghui Zhang:** conceptualization (lead), data curation (lead), formal analysis (lead), funding acquisition (lead), investigation (lead), methodology (lead), project administration (lead), resources (lead), software (lead), supervision (lead), validation (lead), visualization (lead), writing – original draft (lead), writing – review and editing (lead). **Yufei Wu:** investigation (supporting), writing – review and editing (supporting). **Liwei Yang:** funding acquisition (supporting), resources (supporting), writing – review and editing (supporting). **Zekun Li:** visualization (supporting), writing – review and editing (supporting). **Zonglei Li:** investigation (supporting), writing – review and editing (supporting). **Yuying Yang:** investigation (supporting), resources (equal), writing – review and editing (supporting). **Shiyue Chen:** conceptualization (supporting), funding acquisition (supporting), resources (supporting), writing – review and editing (supporting). **Enfeng Liu:** conceptualization (supporting), resources (supporting), visualization (supporting), writing – review and editing (supporting).

## Conflicts of Interest

The authors declare no conflicts of interest.

## Supporting information


Figure S1.

Data S1.

## Data Availability

The data that support the findings of this study are openly available in the Supporting Informations.
