# Supplementary material for: Tracing Aquatic Macrophyte Development in Nansi Lake, Northern China's Largest Freshwater Lake: Plant Macrofossils From 1855 to Present
Source: Ecol Evol. 2025 Jan 23;15(1):e70878. doi: 10.1002/ece3.70878 (PMC11755067; doi:10.1002/ece3.70878)
Supplement: Supplementary file 1 — Figure S1. [file ECE3-15-e70878-s002.doc]

**Supporting Information for:**

Tracing Aquatic Macrophyte Development in Nansi Lake, Northern China's Largest Freshwater Lake: Plant Macrofossils from 1855 to Present

Qinghui Zhang^1*^, Yufei Wu^1^, Liwei Yang^2^, Zekun Li^1^, Zonglei Li^3^, Yuying Yang^1^, Shiyue Chen^2*^, Enfeng Liu^1^

^1^College of Geography and Environment, Shandong Normal University, Ji'nan 250014, China

^2^School of City and Environment, Jiangsu Normal University, Xuzhou 221116, Jiangsu, China

^3^Academic Affairs Division of Xuzhou College of Industrial Technology, Xuzhou 221140, China

***Correspondence**

Qinghui Zhang, College of Geography and Environment, Shandong Normal University, Ji'nan 250014, China. Email: qhzhang@sdnu.edu.cn;

Shiyue Chen, School of City and Environment, Jiangsu Normal University, Xuzhou 221116, Jiangsu, China. Email: [chenshiyue@jsnu.edu.cn](mailto:chenshiyue@jsnu.edu.cn).

**Supplemental figures:** Light microscopy pictures of major aquatic plant remains and animal remains in the sediment core from Weishan Lake. The images are displayed with a grid scale of 1x1mm for reference.


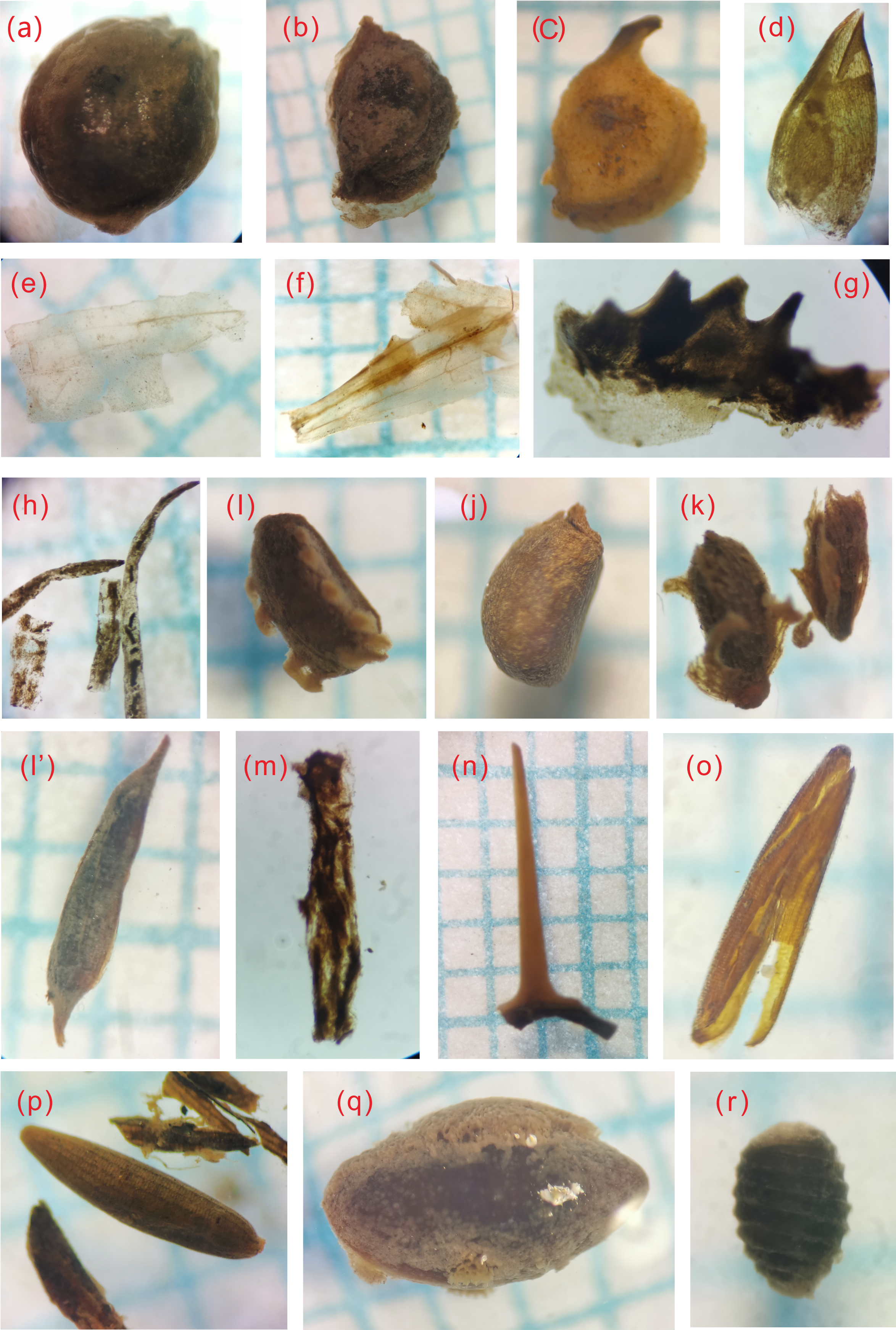


**Figure S1** (a) *Potamgeton lucens* seeds; (b) *Potamogeton malaianus* seeds; (c) *Potamogeton crispus* seeds; (d) *Potamogeton crispus* seed hoods; (e-f) *Potamgeton* leaf fragments; (g) *Potamogeton crispus* turion teeth; (h) *Myriophyllum spicatum* leaf fragments; (i) *Myriophyllum spicatum* seeds; (j) *Myriophyllum verticillatum* seeds; (k) *Vallisneria spinulosa* seeds; (l’) *Hydrilla verticillata* seeds; (m) *Ceratophyllum demersum* leaf spines; (n) *Ceratophyllum demersum* seed spines; (o-p) *Najas minor* seeds; (q) *Najas marina* seeds; (r) *Chara* sp. Oospores


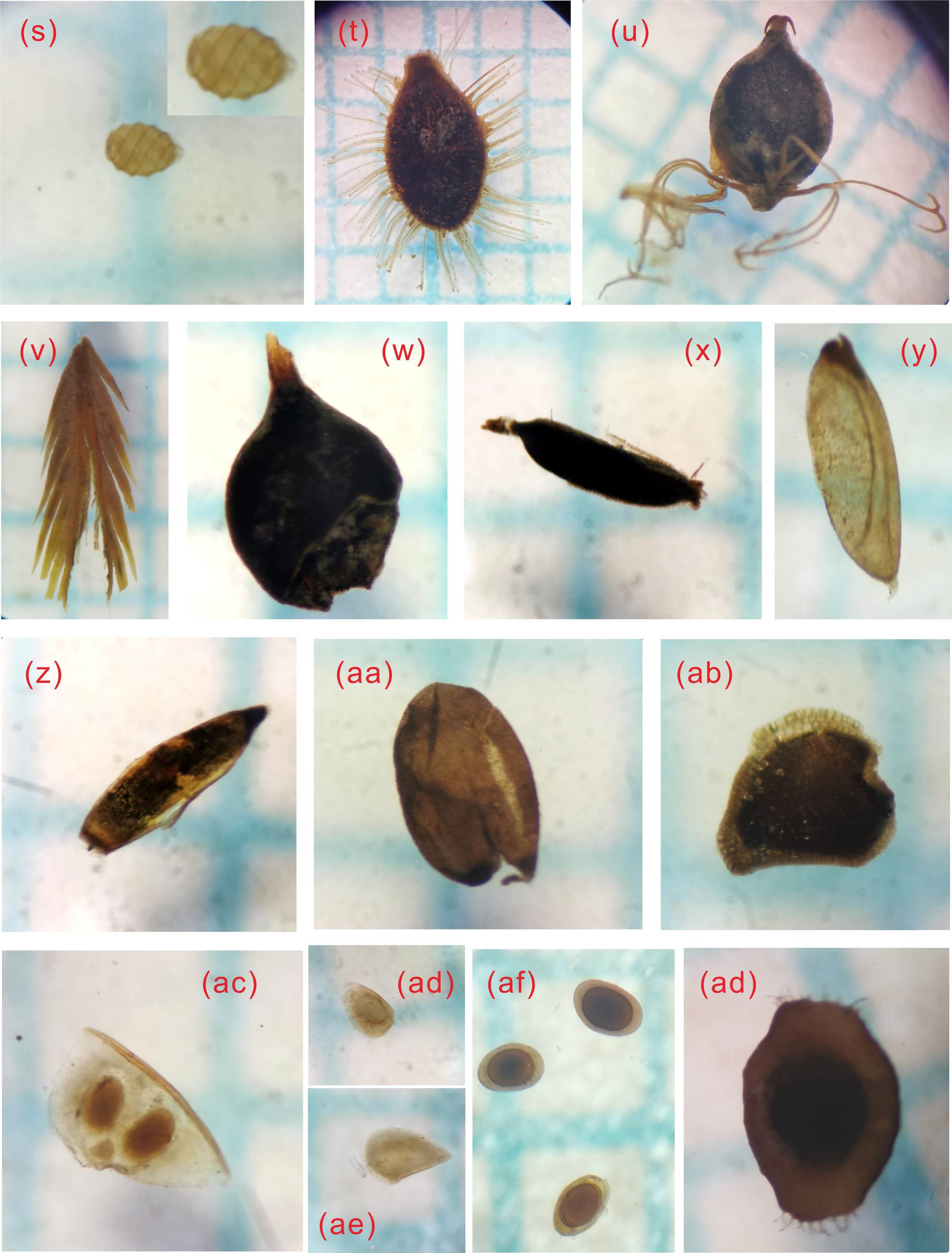


**Figure S2** (s) *Nitella* sp. Oospores; (t) *Nymphoides peltatum* seeds; (u) Polygonaceae seeds; (v) *Trapa natans* barbed appendage of seeds; (w-x) Cyperaceae seeds; (y) *Phragmites australis* seeds; (z) *Typha* sp. seeds; (aa) *Alisma plantago*-aquatica seed; (ab) *Rorippa islandica* seeds; (ac-ad) Cladocera ephippia; (af) *Plumatella* statoblast; (ad) *Lophopodella* statoblast
